# Protocol for the synthesis and activation of hydrogels with photocaged oligonucleotides

**DOI:** 10.1016/j.xpro.2024.103502

**Published:** 2024-12-17

**Authors:** Danielle Herubin, Amanda Yang, Saanvi Gaddam, Katelyn Mathis, Brian Meckes

**Affiliations:** 1 Department of Biomedical Engineering, University of North Texas, Denton, TX 76207, USA; 2 Biodiscovery Institute, University of North Texas, Denton, TX 76205, USA; 3 Texas Academy of Mathematics and Science, University of North Texas, Denton, TX 76205, USA

**Keywords:** Tissue Engineering, Biotechnology and bioengineering, Chemistry, Material sciences

## Abstract

Hydrogels with spatial-temporal control over chemical and physical properties allow for the creation of cellular niches with controllable properties that better mimic tissue environments. Here, we present a protocol for synthesizing hydrogels incorporating photocaged oligonucleotides that can be activated with non-ultraviolet (UV) wavelengths. We detail the synthesis of bulk hydrogels and spatially defined hydrogels with different chemical functionalities that all share common photocaged DNA. Furthermore, we describe conditions for activating photocaged DNA to capture complementary oligonucleotides.

For complete details on the use and execution of this protocol, please refer to Mathis et al.^1^

## Highlights

• Describes the process for creating hydrogels containing photocaged DNA
• Comprehensive instructions for the synthesis of bulk and multifunctional hydrogels
• Details the spatially localized photoactivation of DNA within hydrogels

## Abstract (editor highlights)

Publisher’s note: Undertaking any experimental protocol requires adherence to local institutional guidelines for laboratory safety and ethics.

## Graphical abstract

**Figure:**
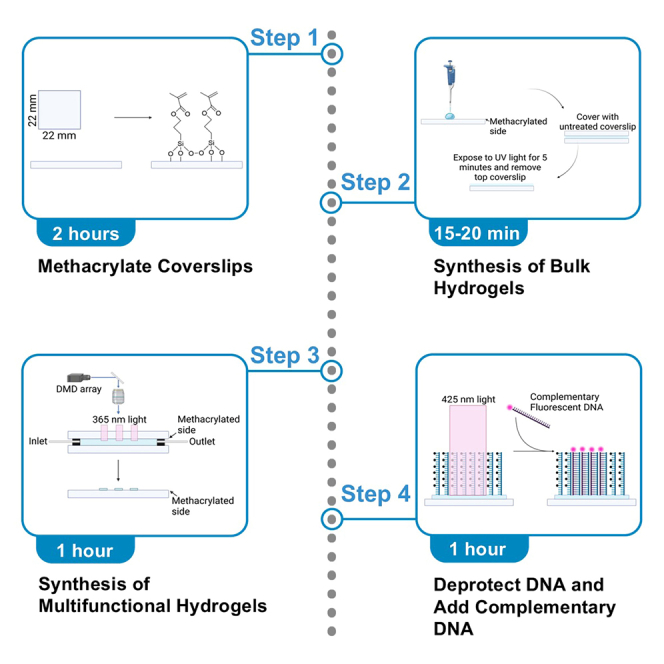

## Before you begin

Photocaged oligonucleotides can be synthesized using standard synthesis procedures, but it is crucial that one minimizes the light exposure. Exposure to ambient conditions does not lead to significant premature decaging; however, foil and/or amber vials should be used in all steps.

### Acrylate functionalization of coverslips

**Timing: 2 h**

**Figure 1:**
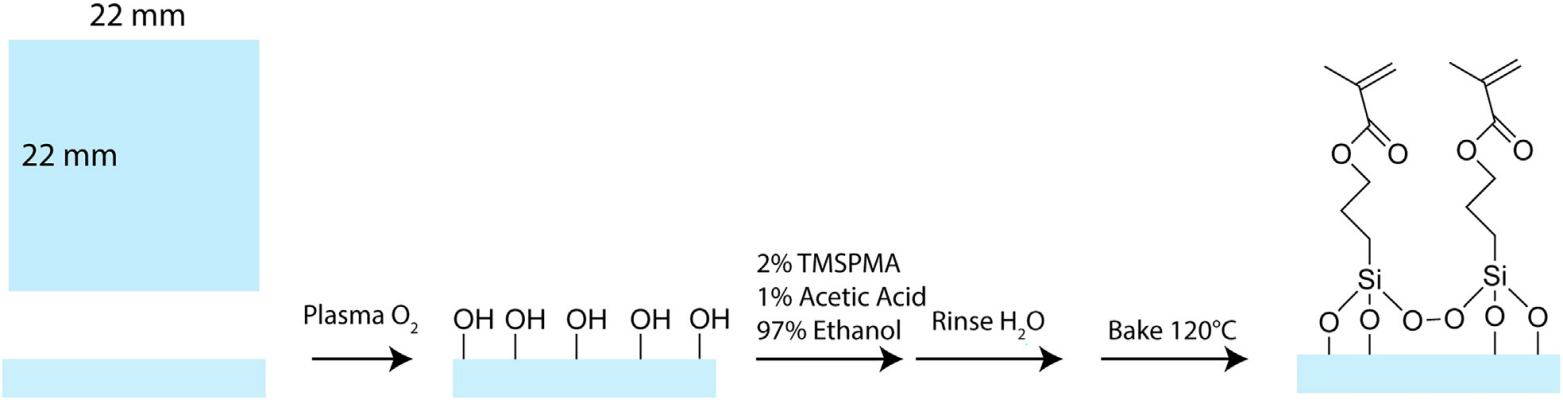
Schematic of methacrylation of glass coverslips Plasma O_2_ treatment of the glass coverslip leads to the formation of hydroxyl groups. In a three-step process, silanes terminated in methacrylate groups are attached to the cover slip.

The following steps will enable the attachment of 3-(Trimethoxysilyl)propyl methacrylate (TMSPMA) to a glass coverslip (#0; 22 × 22 mm; Figure 1). Methacrylation of the coverslip facilitates adhesion between our polyethylene diacrylate (PEGDA) hydrogels and the coverslip. Failure to perform this procedure will result in the release of gels from the coverslip and prevent multifunctional printing. This protocol is based on steps first reported by.^2^

1. Place coverslips in a small weighing boat or 50 mL tube and cover in a 70% (v/v) ethanol solution.
2. Place the weighing boat in a bath sonicator and sonicate coverslips.
  a. Sonicate without heat for 30 min.
3. Air-dry coverslips by flowing air over the top for a couple of seconds and then placing coverslips in six-well plates.
4. Oxygen plasma etch (PE-25 series, Plasma Etch Inc) coverslips for 5 min.
  a. Adjust the O_2_ volume flow rate on plasma etch between 7-12 cc/min.
  b. Set pressure below 200 mTorr before plasma initiation.
5. Place coverslips in 2% (v/v) TMSPMA solution for 5 min.
  a. The 2% (v/v) TMSPMA solution contains ethanol (19.4 mL), glacial acetic acid (200 μL), and 3-(Trimethoxysilyl)propyl methacrylate (400 μL).
  b. Rinse thoroughly under DI water.
6. Place coverslips in an oven (Thermocenter, Salvis) for 1 h at a temperature of 120°C.
7. Store coverslips under vacuum for up to one month.

### Synthesis of oligonucleotides

**Timing: 1 day**

The PEGDA hydrogels used in this study feature photocaged oligonucleotides that contain guanines caged by 7-(diethylamino)coumarin-4-yl]-methyl (DEACM, a bulky coumarin-derivative) that prevent complementary DNA from binding until the DEACM groups are cleaved away by light.^3^ A critical number of photocaged bases (5 to 6 per 20 bases) are required to prevent hybridization.^4^^,^^5^

8. Weigh out CPGs for synthesis
  a. Weights vary depending on the synthesis scale and the exact properties of the batch of CPG. The label provides the molar-to-weight ratio in μmol/mg.

***Note:*** For gel linker strands (Hydrogel linker DNA), use the 1-O-Dimethoxytrityl-propyl-disulfide,1′-succinyl-lcaa-CPG.

9. Load CPG and reagents into the DNA synthesizer and input the appropriate sequences for the DNA you are making.

***Note:*** Sequences are provided in the key resources table.

***Note:*** Ensure the synthesis is performed with the trityl-on to facilitate purification.

***Note:*** We use a 5-minute coupling time for the DEACM phosphoramidites to increase yields.

10. Dry column containing CPGs under air or argon.
11. Transfer the CPGs to a glass vial and add 1 mL of saturated ammonium hydroxide (28–32%) at 20°C–25°C overnight (16–18 h)

**CRITICAL:** Ensure that the ammonium hydroxide is fresh to achieve complete deprotections.

12. Remove ammonium hydroxide by placing the sample in the Multivap (Organomation) and flowing nitrogen/air over the sample at 10–20 L/min (1–2 h).

### Purification of DNA

**Timing: 1–2 days**

The purification of DNA removes any failure sequences from the final solution and removes the trityl protection group from the 5′ end of the DNA.

13. Add 1 mL of 100 mg/mL Sodium Chloride solution to the deprotected oligonucleotides suspended in 1 mL of H_2_O for a final volume of 2 mL.
14. Condition a Glen-Pak DNA purification cartridge with 0.5 mL of acetonitrile and 1 mL of 2.0 M triethylamine acetate.
  a. Inject the reagents into the cartridge by connecting a Luer-Lok syringe to the top of the cartridge and pushing the reagents through.
15. Inject 1 mL of oligonucleotide/salt mixture into the cartridge. Repeat this step until all of the solution has been injected.
16. Rinse the cartridge with 2 mL of 5% (v/v) acetonitrile in 100 mg/mL sodium chloride.
17. Detritylate the DNA with 2 mL of 2% (v/v) trifluoroacetic acid.
18. Rinse the cartridge with 2 mL of deionized water.
19. Wash DNA from the column with a 1 mL injection of 50% (v/v) acetonitrile in DI water containing 0.5% (v/v) ammonium hydroxide and collect the eluent in a 15 mL Falcon tube as it flows out.
  a. Repeat the injection to ensure that all DNA is recovered.
20. Freeze the collected eluent.
21. Place frozen liquid with DNA in a lyophilizer. Leave it for 24–48 h.
22. DNA can then be reconstituted in DI water (1 mL) and placed in a −80°C freezer.
23. Determine the yield of DNA and dilute out to a final concentration of 200 μM.
  a. Measure the absorbance at 260 nm with a UV-Vis spectrometer.
  b. Use the equation below to calculate the concentration. A260 = the measured absorbance at 260 nm. DF = dilution factor. E = extinction coefficient (this can be found using OligoAnalyzer (IDT) software).
  c. If the concentration is less than 200 μM, you can lyophilize again and reconstitute with less DI water.

***Note:*** If you are making the thiol DNA (Hydrogel linker DNA), a further step is necessary to cleave the disulfide group on the 3′ end into a thiol group.

24. Cleave disulfide bonds on the 3′ end of the DNA.
  a. Add 50 μL of 100 μM tris(2-carboxyethyl)phosphine hydrochloride (TCEP) into 950 μL of DNA solution.
  b. Shake for 5 min.
  c. Add 50 μL of 1X PBS.

$$C\;\left(\mu M\right)=A260\times DF\times 10^{6}÷E\;\left(\frac{L}{mol\cdot cm}\right)$$

### Microscope configuration and focal length corrections

**Timing: 15 min**

Prior to starting experiments, a microscope must be configured to allow for spatially controlled printing. Below, we describe how our microscope is configured and the key components in our system that enable printing on an Olympus BX63 microscope. However, the protocol can operate with any microscope, although a motoroized system will make this process easier. We have included the optical setup we use in combination with our microscope that supports three different wavelengths to the digital micromirror device (DMD; Polygon 1000, Mightex) connected to the microscope for patterning experiments. The key component to our configuration is that we have a liquid light guide connecting a 365 nm (Sutter Instruments), 455 nm (Thorlabs), and 617 nm (Thorlabs) LED to our DMD. The 617 nm light can be used to focus light without damaging photoresponsive moieties (either prematurely print hydrogels or activate oligonucleotides). We use the 365 nm and 455 nm wavelengths for the photochemistry experiments in this protocol for hydrogel synthesis and DNA activation, respectively. These wavelengths will often have different focal planes than the 617 nm LED used for positioning. On most systems, this can be adjusted before use. Here, we outline steps to check differences in focal planes that can be adjusted automatically on motorized microscopes.

25. Place a coverslip on the microscope stage.
26. Load a pattern to the Polygon 1000 in the Polyscan2 software.
27. Turn on the 617 nm LED connected to the Polygon 1000.
28. Adjust the z-plane until a crisp image comes into view.
29. Record the Z-position value when the light pattern is in focus on the sample.
30. Turn off the 617 nm LED.
31. Turn on the 365 nm LED.
32. Adjust the Z-position until the microscope displays a crisp image of the shape.
33. Record the Z-position of the in-focus pattern.
34. Subtract the Z-plane value of the 617 nm laser from the Z-plane value of the 365 nm laser to calculate the difference in the focal lengths of the two lasers.
35. Repeat steps 30–34 for the 455 nm LED light source.

***Note:*** This calculated value should be recorded and used to make corrections during printing. If using CellSens, this should be used as the “Move-Z” value or by entering an offset between image channels.

## Key resources table

**Table:** 

REAGENT or RESOURCE	SOURCE	IDENTIFIER
**Chemicals, peptides, and recombinant proteins**		

Orange G	Thermo Scientific Chemicals	Cat#416550100
Polyethylene glycol diacrylate	Sigma-Aldrich	CAS:26570-48-9 Cat# 455008
Diphenyl(2,4,6-trimethylbenzoyl)phosphine oxide	Sigma-Aldrich	Cat#906808
3-(trimethoxysilyl)propyl methacrylate	Sigma-Aldrich	Cat#440159
5′-dimethoxytrityl-N2-(4-isopropylphenoxyacetyl)-O6-[[7-(diethylamino)coumarin-4-yl]-methyl]-2′-deoxyGuanosine, 3'-[(2-cyanoethyl)-(N,N-diisopropyl)]-phosphoramidite (DEACM-phosphoramidite)	Glen Research	Cat#10-1533-90E
1-O-dimethoxytrityl-propyl-disulfide,1′-succinyl-lcaa-CPG	Glen Research	Cat#20-2933-10
Polyethylene glycol thiol (1 kD)	Creative PEGWorks	Cat#PLS-606
2.0 M triethylamine acetate	Glen Research	Cat#60-4110-57
2% trifluoroacetic acid	Glen Research	Cat#60-4040
1x PBS	Corning	Cat#21-040-CV
Saturated ammonium hydroxide (28%–32%)	Fisher Chemical	Cat#A669-500
Anhydrous acetonitrile	Glen Research	Cat#40-4050-45
Glen-Pak DNA purification column	Glen Research	Cat#60-5200-10
tris(2-carboxyethyl)phosphine hydrochloride	Sigma-Aldrich	Cat#51805-45-9
Glacial acetic acid	Fisher Chemical	CAS: 64-19-7
Ethanol	Fisher Chemical	CAS: 64-17-5
DuPont MOLYKOTE vaccum grease	Structure Probe Inc	Cat# 05054-AB
Sodium chloride	Fisher Bioreagents	CAS: 7647-14-5

**Oligonucleotides**		

ACAACAACAAGCAACAACAA TTT TTT TTT TTT TTT TTT TT-Thiol	custom synthesis	Hydrogel linker DNA
T∗T∗G TTG TTG CTT GTT GTT GTA DAA DAA DAA CGA ADA ADA∗ A∗ (Note: D = DEACM caged dG-CE, X∗ = phosphorothioate backbone)	custom synthesis	PC-DNA
T∗T∗CTTCTTCGTTCTTCTTC∗T∗Cy5	custom synthesis	Probe Oligo 1
T∗T∗CTTCTTCGTTCTTCTTC∗T∗Cy3	custom synthesis	Probe Oligo 2

**Software and algorithms**		

cellSens	Olympus	https://www.olympus-lifescience.com
PolyScan3	Mightex	https://www.mightexbio.com
OligoAnalyzer Tool	IDT	https://www.idtdna.com/pages/tools/oligoanalyzer

**Other**		

BX63 microscope	Olympus	https://www.olympus-lifescience.com
Polygon 1000	Mightex	https://www.mightexbio.com
Thermocenter	SalvisLab	https://salvislab.com
−105°C lyophilizer	Labconco	https://www.labconco.com
MULTIVAP	Organomation	https://www.organomation.com
Expedite DNA synthesizer	Biolytic	https://www.biolytic.com
Perfusion chamber	Warner Instruments	Cat#RC21B
PE-25 series plasma system	Plasma Etch, Inc.	https://www.plasmaetch.com
365 nm Lambda FLED	Sutter Instrument	https://www.sutter.com
617 nm LED	Thorlabs	Cat#M617L3
455 nm LED	Thorlabs	Cat#M455L4
#0, 22 × 22 mm coverslips	Ted Pella	Cat#260300
Hamilton 1000 series, luer lock syringe	Sigma-Aldrich	Cat# 26208

## Materials and equipment

**Table:** Polymer precursor solution 1 for bulk hydrogels

Reagent	Concentration	Amount
Polyethylene Glycol Diacrylate (PEGDA)	-	18 μL
Water-soluble TPO-based nanoparticle photoinitiator	100 mg/mL	30 μL
Polyethylene Glycol Thiol (PEG-SH)	50 mg/mL	45 μL
Thiol DNA (Hydrogel linker DNA)	200 μM	125 μL
Deionized (DI) Water	-	32 μL
**Total**	**N/A**	250 μL

***Note:*** This solution cannot be kept overnight. The stock solution of TPO will last for weeks at 2°C–4°C, but the PEG-SH stock solution must be fresh.

**Table:** Polymer precursor solution 2 for patterning hydrogels

Reagent	Concentration	Amount
PEGDA	-	72 μL
Water-soluble TPO-based nanoparticle photoinitiator	100 mg/mL	120 μL
Polyethylene Glycol Thiol (PEG-SH)	50 mg/mL	180 μL
Thiol DNA (Hydrogel linker DNA)	200 μM	500 μL
Orange G	100 mg/mL	10 μL
Deionized (DI) Water	-	98 μL
**Total**	**N/A**	980 μL

***Note:*** This solution cannot be kept overnight. The TPO stock solution will last for weeks at 2°C–4°C, and a stock Orange G solution can be made and kept at 20°C–23°C for weeks, but the PEG-SH stock solution must be made fresh.

***Alternatives:*** Microscopy: Any upright microscope can be used in this configuration. The accompanying software will also change to one compatible with the brand chosen.

***Alternatives:*** Digital micromirror device: Any brand of DMD can be used. The software for controlling the mirrors may change (although Polyscan2 software is compatible with different micromirror devices).

***Alternatives:*** DNA synthesis: All DNA was synthesized in-house using a Biolytic DNA synthesizer and Glen Research reagents. Any synthesizer brand can be utilized to create the oligonucleotides along with any reagent brands. Only the DEACM-caged oligonucleotides must be used along with the prescribed deprotection schemes and coupling times.

## Step-by-step method details

### Synthesis of bulk hydrogels with photocaged oligonucleotides

**Timing: 15–20 min**

**Figure 2:**
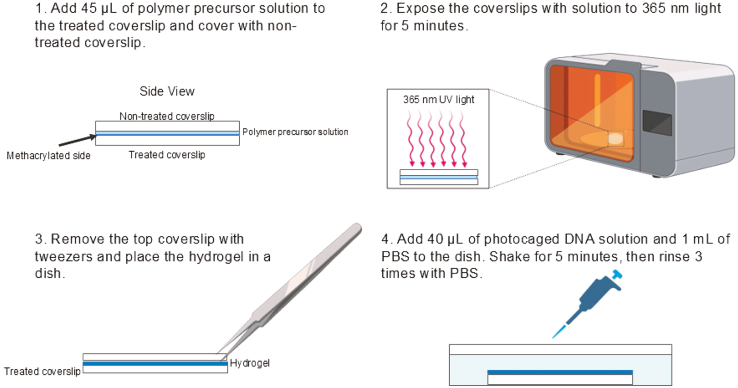
Schematic for formation of hydrogels with bulk properties that contain photocaged oligonucleotides

This step details the creation of hydrogels with homogenous bulk properties that contain photocaged oligonucleotides (Figure 2). These hydrogels are formed via a thiol-ene click reaction and contain a combination of polyethylene glycol (PEG)-thiols and polyethylene diacrylate (PEGDA). The ratios we utilize are based on those previously reported.^6^ We include DNA with 3′ thiols to allow them to readily crosslink into the hydrogels. The stiffness of the hydrogels can be tuned by controlling the PEGDA amount in the solution. Increasing PEGDA increases the stiffness.^1^ If one wants to create hydrogels with spatially defined properties, skip steps 1–8 and proceed to step 9.

1. Place the functionalized coverslips facing up on individual petri dish plates.
2. Add 45 μL of the polymer precursor solution 1 (Table 1) onto each plasma-etched glass coverslip with the side facing up.
3. Place a new non-treated coverslip on top of the solution, creating an even layer of hydrogel.
4. Expose the coverslips with the solution to 365 nm light for 5 min to form a hydrogel.
5. Remove the petri dishes with the hydrogels from the UV machine and take the top coverslip off the hydrogels using tweezers.
6. Cover each remaining coverslip with 1 mL of 1X PBS (144 mg/L potassium phosphate monobasic, 9000 mg/L sodium chloride, and 795 mg/L sodium phosphate dibasic).
7. Add 40 μL of 200 μM Photoprotected Adaptor DNA (PC-DNA) to each coverslip with the PBS and shake for 5 min.

***Note:*** Complete this and the following steps in the dark.

8. Cover the petri dish plates in aluminum foil and place them on a shaker for 5 min to protect them from light and prevent unwanted degradation.
9. Rinse with 1 mL 1X PBS 3 times for 5 min each and rinse to remove excess DNA that did not attach to the hydrogels.

### Synthesis of hydrogels with unique chemical properties

**Timing: 1 h**

Create small hydrogels with features ranging from cell scale to hundreds of μm, where different gels on a coverslip contain unique chemical compositions. The chemical functionality is controlled by adding different thiol-containing moieties to the hydrogels. This step is completed in place of creating bulk hydrogels (steps 1–8).

10. Make 2 vials of the polymer precursor solution 2 (Table 2).
  a. Add 20 μL of rhodamine-PEG-thiol (10 μM) to one vial
  b. Add 20 μL of biotin-PEG-thiol (10 μM) to a second vial.

***Note:*** Many different thiol moieties can be added to the gel and reacted at this time.

**Figure 3:**
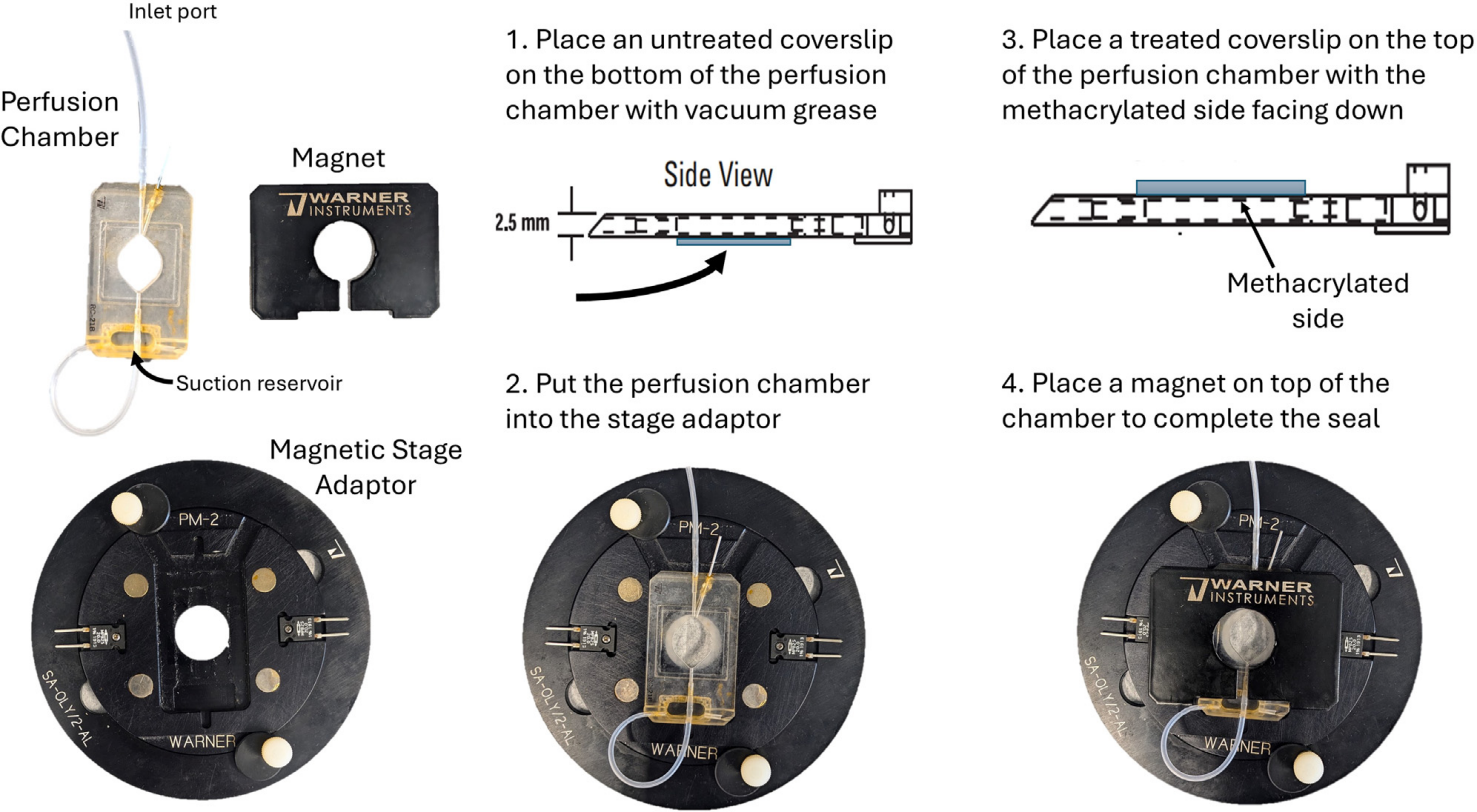
Schematic for setup of the perfusion chamber The coverslips are placed on both sides of the perfusion chamber in order to create a seal to retain the liquid. The perfusion chamber is placed on a magnetic stage adaptor for use with the microscope. A magnet is placed on top to complete the seal and keep the components in place.
12. Inject the rhodamine-containing precursor solution with a syringe into the inlet port of the perfusion chamber until the solution starts dripping into the suction reservoir.

***Note:*** Do not take the syringe out of the inlet port.

11. Set up a methacrylated coverslip in a perfusion chamber (RC21B; Warner Instruments) with the methacrylated side facing down (Figure 3).
  a. Prepare the chamber by applying a small amount of vacuum grease to the square outline on the bottom of the chamber.
  b. Place an untreated coverslip on the grease and press firmly to ensure the solution will not leak out when injected into the chamber.
  c. Put the chamber into a magnetic stage adaptor (Warner instruments).
  d. Place the methacrylated coverslip on the top side of the chamber within the square outline, with the treated side facing down.
  e. Place the magnet on top of the chamber to complete the seal.

**CRITICAL:** Make sure there are no bubbles within the chamber while filling.

**CRITICAL:** No hydrogels will form if the precursor solution does not touch the top coverslip.

***Note:*** Work quickly after this step, as the solution will evaporate and form bubbles.

**Figure 4:**
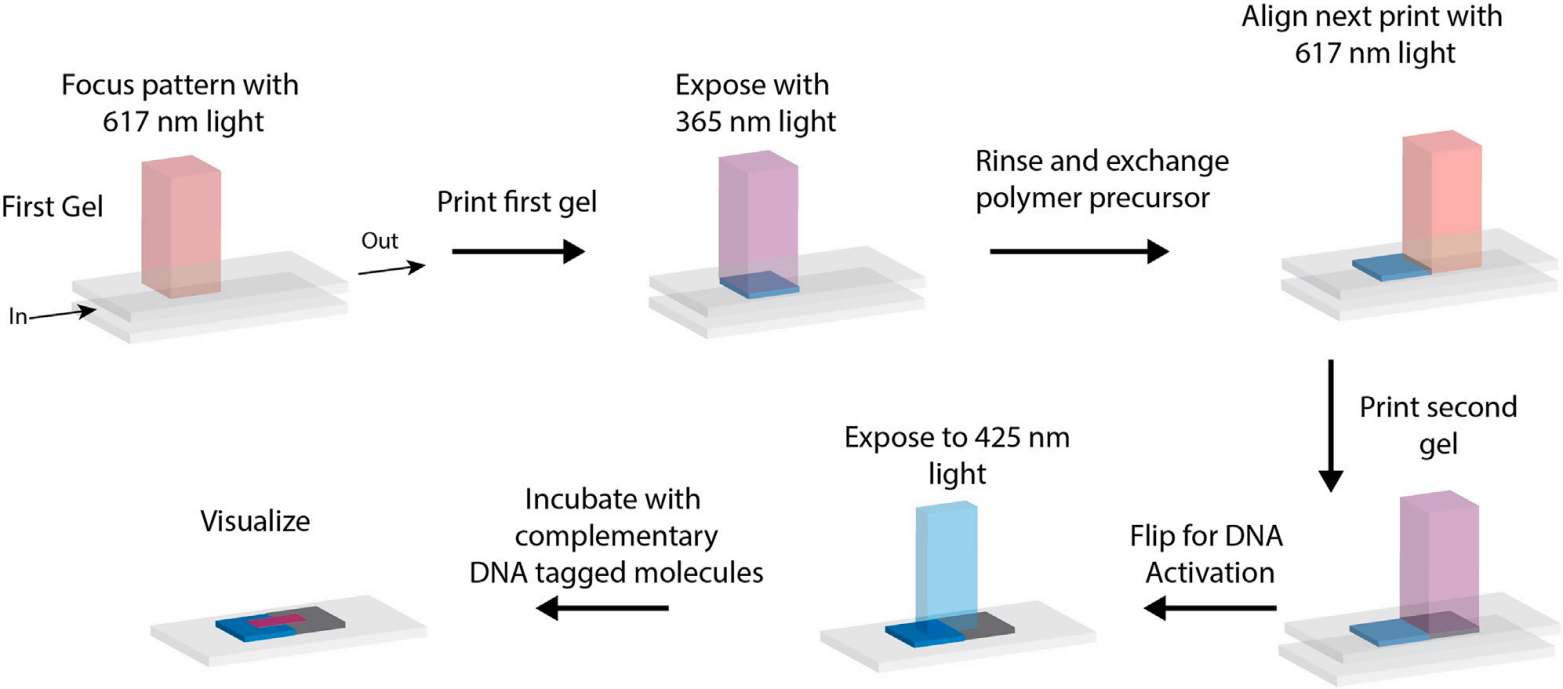
Schematic of printing steps for formation of hydrogels with unique chemical properties The first precursor solution is injected into the perfusion chamber. A specific area is exposed to 365 nm light using a DMD to polymerize the gel. The first solution is rinsed and exchanged with the second precursor solution. The DMD pattern is aligned next to the first gel; the second gel is polymerized with 365 nm light. The gels are then exposed to 425 nm light to decage the DNA and then incubated with fluorescent complementary DNA.

***Note:*** The exact wavelength of this laser is not important as long as it is outside of the range that will activate the photoinitiator (>500 nm).

13. Set up the DMD to shine a 615 nm laser in desired areas (Figure 4).

14. Focus the projected DMD light on the liquid-cover slip interface.
  a. Adjust the z-plane of the objective/sample until the image is crisp.

**CRITICAL:** There is often a difference in focal lengths for the 615 nm laser and the 365 nm laser. Ensure that the pattern is in focus when the 365 nm laser is on and has been properly calibrated before starting the experiment. Focus the 365 nm laser in a corner of the sample that will not be used for patterning to prevent premature activation of the photoinitiator.

15. Expose for 20–30 s.

***Note:*** Exposure times can vary depending on the light intensities and optical path properties. Exposing for times ranging from 5 to 45 s at 5 s intervals will allow one to determine the best setting for their system.

16. Rinse with DI water by injecting 5 mL through the perfusion chamber.
17. Inject the biotin-containing precursor solution into the perfusion chamber.
18. Move the microscope stage so that the laser will shine on a different coverslip area.
  a. Use the 617 nm LED to guide your location.
19. Focus the projected DMD light on the liquid-cover slip interface.
  a. Adjust the z-plane of the objective/sample until the image is crisp.
20. Expose with 365 nm light for 20–30 s.
21. Rinse with 5 mL DI water.

***Note:*** Steps 17–21 can be repeated with different precursor inks as desired.

22. Take the top coverslip out of the perfusion chamber and flip it upside down before placing it into a dish.
23. Cover the hydrogel with 1 mL PBS.
24. Add 40 μL of 200 μM Photoprotected Adaptor DNA (PC-DNA) to each cover slip with the PBS.

***Note:*** Complete this and the following steps in the dark to prevent light exposure.

25. Cover the petri dish plates in aluminum foil and place them on a shaker for 5 min to protect them from light and prevent unwanted degradation.
26. Rinse with 1 mL PBS 3 times for 5 min each and rinse to remove excess DNA that did not attach to the hydrogels.

### De-caging of oligonucleotides and capture of complementary strands

**Timing: 1 h**

We describe the use of a DMD to decage oligonucleotides to allow for localized capture of DNA. DEACM photocages allow for activation outside the UV range,^3^^,^^7^ potentially enhancing biocompatibility.

27. Place a coverslip with a hydrogel facing up in a petri dish. Put the dish on the microscope stage.

***Note:*** Ensure the hydrogel is covered with 1X PBS.

28. Set up the DMD to shine light in the desired pattern shapes.
29. Use the 617 nm LED to focus your pattern on the desired area of the hydrogel.
  a. Focus the projected DMD light on the hydrogel surface (the second focal plane from the top).

***Note:*** The exact wavelength of this light source is not important as long as it is outside of the range that will cleave the DEACM groups (>505 nm).

30. Switch to a 425 nm LED (ThorLabs, power of 1445 mW) and expose for 1 min.

***Note:*** The following steps detail capture of fluorophore probes. Other functionalities can be substituted as desired. Steps 31– 37 can be substituted for other functionalities/skipped depending on the application.

31. Remove the PBS. Incubate the gel with 2 μL Cy5-labeled complementary DNA (Probe Oligo 1) and 1 mL PBS for 1 min, and then rinse 3 times with 1 mL PBS.
  a. Rinse by slowly adding PBS to the side of the dish, wait 1 min, and then carefully remove the PBS.
32. Cover the hydrogel with PBS.
33. For additional patterning steps, repeat steps 1-4 to decage the DNA in a different area of the hydrogel.
34. Remove the PBS. Incubate the gel with 2 μL Cy3-labeled complementary DNA (Probe Oligo 2) and 1 mL PBS for 1 min and then rinse 3 times with 1 mL PBS.
  a. Rinse by slowly adding PBS to the side of the dish, wait 1 min, and then carefully remove the PBS.
35. Cover the hydrogel with PBS.
36. Select your desired objective and filter on your microscope setup.
37. Image on the correct channel for your employed fluorophore.

## Expected outcomes

**Figure 5:**
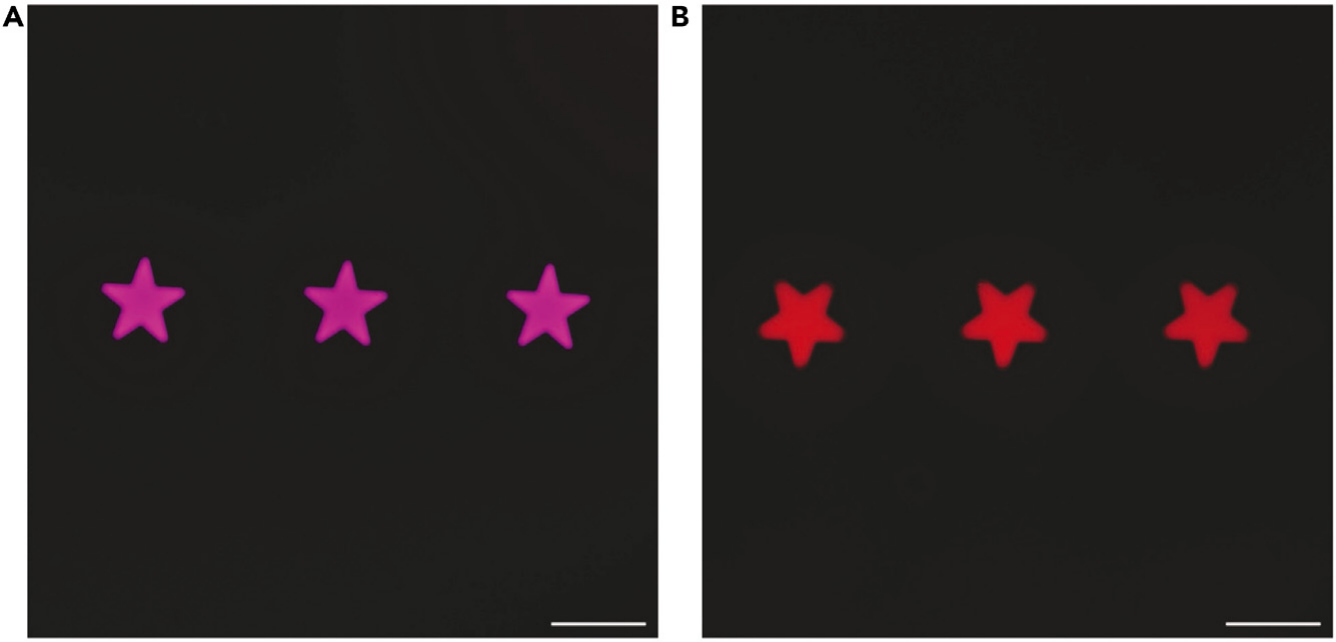
Fluorescence micrographs of DNA patterns on hydrogels with bulk properties (A) Cy5 channel showing Cy5-labeled complementary DNA. (B) Cy3 channel showing Cy3-labeled complementary DNA. Scale bars = 500 μm.

**Figure 6:**
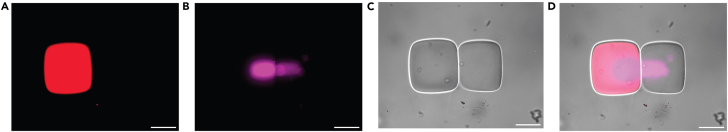
Fluorescence micrographs of hydrogels with unique chemical properties (A) TX RED channel shows the hydrogel containing rhodamine only. (B) Cy5 channel shows the Cy5-labeled complementary DNA. (C) Bright-field image shows both gels next to each other. (D) Merge of all channels. All scale bars = 200 μm.

This protocol allows one to create hydrogels that contain photocaged oligonucleotides. Importantly, the synthesis procedures presented in this manuscript detail how to create bulk hydrogels and how one can print hydrogels with different chemical functionalities that have common photocaged oligonucleotides (Figures 5 and 6). The photocages on the DNA can be removed using non-UV light to allow one to capture target moieties attached to DNA that is complementary to the photocaged DNA. The protocol also details how uncaging of the DNA in the hydrogels can be done locally using a DMD device to shape light (Figures 5 and 6). While the theoretical size limit for the activation of the DNA is the diffraction of light, practically, it will also depend on dispersion in media and the magnification of the objective used. We have regularly achieved features with 10 μm resolution. Importantly, we can repeatedly address hydrogels with light to decage DNA in different locations to capture different functionalities, as shown by our prints containing different fluorophore-labeled oligonucleotides. Mathis et al. demonstrate the localized capture of cells that can be achieved by incubating decaged hydrogels with cells coated with complementary DNA.^1^ Overall, the completion of this protocol results in hydrogels with spatially and temporally tunable properties that can be accessed across hydrogels with different chemical functionalities.

## Limitations

The printing resolutions will be defined by a combination of the wavelengths of the light, the exposure time, and the pixel size of the DMD. In addition, changes to certain parts of the composition of the hydrogels are likely to impact components of the material properties that may be unpredictable if they significantly change the polymerization process. We have included troubleshooting steps and recommendations for parameters to test on a different system.

## Troubleshooting

### Problem 1

Hydrogels only weakly attach to the surface of the coverslip (related to the steps taken before you begin steps 1–7 and steps 1–23 of the step-by-step method details).

### Potential solution

• Coverslips have not been properly methacrylated, or the surface coating has degraded: Coat new coverslips with TMSPMA. Ensure all reagents remain fresh.

### Problem 2

No fluorescence appears in the hydrogel after performing decaging steps when imaging with a microscope in step 33.

### Potential solution

• Insufficient energy for decaging the oligonucleotides: Test multiple exposure times to ensure sufficient energy has been introduced to the surface or increase the light intensity. As a structured approach, one can systematically test different exposure times. We recommend that one attempts 1, 5, 15, 30, 45, 60, and 120 s as exposure times with maximum brightness set for the LED. After incubating with fluorophore-labeled DNA and rinsing (steps 30–36), one can image the resulting patterns and observe when the patterns no longer get brighter, indicating that full decaging has occurred. The goal is to fully decage the DNA.
• Oligonucleotide degradation: Check oligonucleotides using a 20% PAGE gel electrophoresis to ensure that hybridization occurs. Perform a control experiment where oligonucleotides are decaged and see if the sequences hybridize with the probe sequence.

### Problem 3

Incomplete polymerization or no polymerization is observed when synthesizing hydrogels. When visualizing the gels, there are no observed patterns (related to steps 9–23 of the step-by-step method details).

### Potential solution

• Too little energy: Most stereolithographic polymerization occurs through a two-step process.^8^ Sufficient energy is required for gelation, followed by polymerization growth. Increase the exposure time to increase the energy dose to the surface or increase the intensity of the light source.
• Reagents have degraded: You can test to ensure polymerization can still occur by mixing the precursor solution in a microcentrifuge tube and exposing the whole tube to UV light.
• No adhesion of the gel to the coverslip: Ensure that the coverslip has been fully methacrylated before printing and that you are printing on the right surface.

### Problem 4

**Figure 7:**
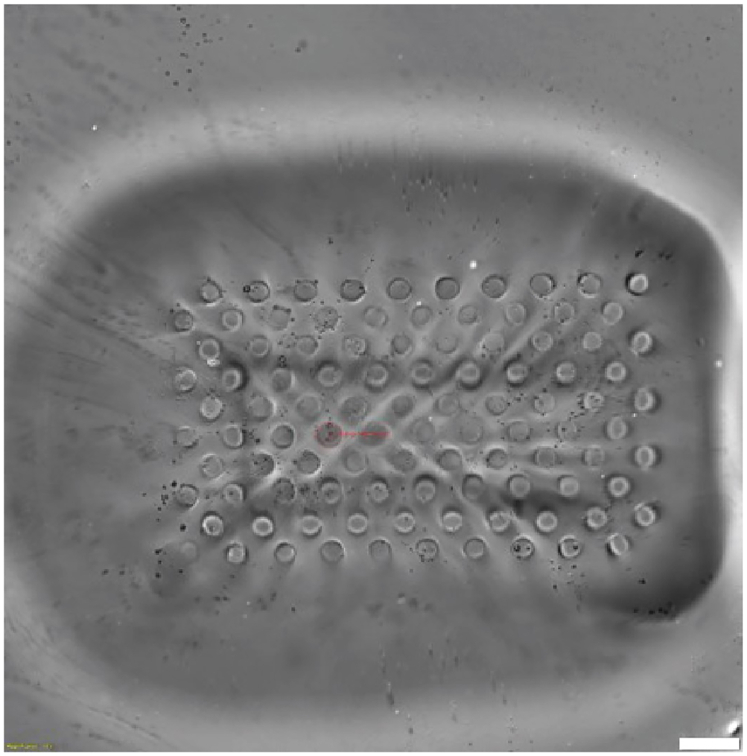
Bright-field images of overpolymerized gels Scale bar = 100 μm.

Overpolymerization of the hydrogels is observed while patterning with a DMD. The patterns are not sharp, or large chunks of polymer are everywhere (steps 9–23 of the step-by-step method details, Figure 7).

### Potential solution

• Too much light energy: Lower the exposure time to the 365 nm light source or decrease the power to this light.
• Improper focus: Ensure that the DMD is focused on the surface and that compensation between different wavelengths is correct.
• Improper ratios: Double-check the ratios of all the polymerization ingredients and ensure they are all still fresh.

### Problem 5

**Figure 8:**
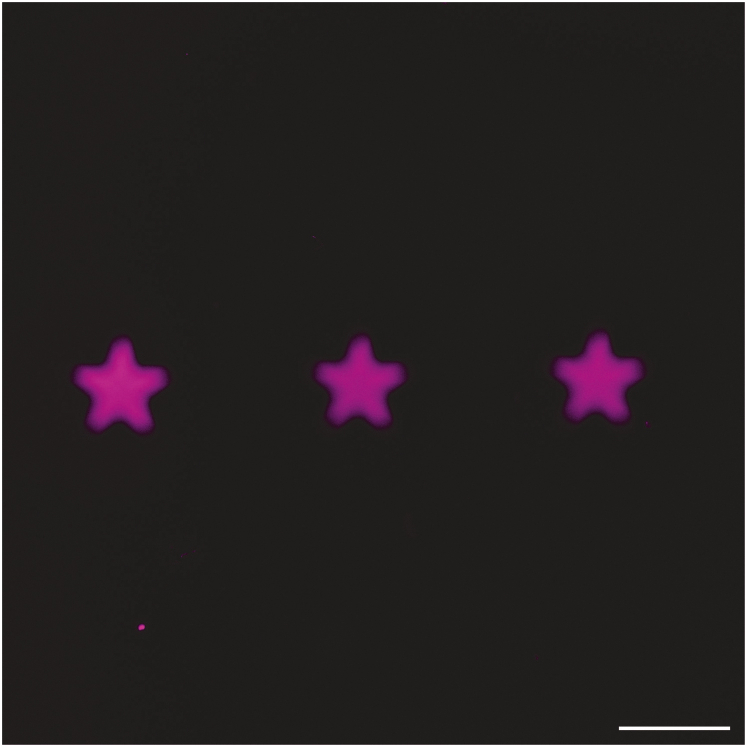
Fluorescence micrograph of out-of-focus DNA patterns on bulk hydrogels These patterns were not in focus, resulting in blurry images. Scale bar = 500 μm.

The DNA patterns are not sharp and appear blurry (related to steps 24–27 of the step-by-step method details, Figure 8).

### Potential solution

• Improper focus: Ensure that the DMD is focused on the surface and that compensation between different wavelengths is correct. There is a difference in focal lengths between the 617 nm laser and the 425 nm laser. Ensure the pattern is in focus once you switch to the 425 nm laser.
• Focus is on the wrong interface: The correct focal plane for patterning should be the hydrogel-liquid interface. The patterning can appear in focus if you are on the hydrogel-coverslip interface, but the resulting DNA patterns will be blurry.
• Insufficient light exposure: Test multiple exposure times to ensure sufficient energy is introduced to the surface or increase the light intensity.

## Resource availability

### Lead contact

Further information and requests for resources and reagents should be directed to and will be fulfilled by the lead contact, Brian Meckes (brian.meckes@unt.edu).

### Technical contact

Technical questions on executing this protocol should be directed to and will be answered by the technical contact, Katelyn Mathis (katelynmathis@my.unt.edu).

### Materials availability

This protocol did not generate any new unique materials. Reagents can be purchased from suppliers noted in the KRT.

### Data and code availability

All data reported in this protocol will be shared by the lead contact upon reasonable request. This protocol does not report original code.

## Acknowledgments

The research reported in this publication was supported by the National Institute of General Medical Sciences of the National Institutes of Health under award R35GM150577 (B.M.). The content is solely the responsibility of the authors and does not necessarily represent the official views of the National Institutes of Health.

## Author contributions

Conceptualization, K.M. and B.M.; methodology, D.H., A.Y., S.G., K.M., and B.M.; investigation, D.H., A.Y., S.G., and K.M.; writing – original draft, D.H., A.Y., S.G., K.M., and B.M.; writing – review and editing, D.H., A.Y., S.G., K.M., and B.M.; funding acquisition, B.M.; resources, B.M.; supervision, K.M. and B.M.

## Declaration of interests

The authors declare no competing interests.
